# Mapping cellular vulnerability in Parkinson’s disease using retro-AAVs and preformed α-synuclein fibrils

**DOI:** 10.1186/s40035-026-00535-7

**Published:** 2026-01-30

**Authors:** Fanni F. Geibl, Ahmed A. S. Musa, Leo Dietrich, Helena Wolter, David L. Wokosin, Sharof Khudayberdiev, Marco B. Rust, Rong Chen, Valina L. Dawson, Ted M. Dawson, Wolfgang H. Oertel, D. James Surmeier, Martin T. Henrich

**Affiliations:** 1 Department of Psychiatry and Psychotherapy, Marburg University, Rudolf-Bultmann-Str. 8, 35043 Marburg, Germany; 2 Department of Neurology, Marburg University, 35043 Marburg, Germany; 3 Department of Neuroscience, Feinberg School of Medicine, Northwestern University, Chicago, IL 60611 USA; 4 Molecular Neurobiology Group, Institute of Physiological Chemistry, Marburg University, 35032 Marburg, Germany; 5 Center for Mind, Brain and Behavior (CMBB), University of Marburg and Justus-Liebig-University Giessen, 35032 Marburg, Germany; 6 Neuroregeneration and Stem Cell Programs, Institute for Cell Engineering, Johns Hopkins University School of Medicine, Baltimore, MD 21205 USA; 7 Department of Neurology, Johns Hopkins University School of Medicine, Baltimore, MD 21205 USA; 8 Solomon H. Snyder Department of Neuroscience, Johns Hopkins University School of Medicine, Baltimore, MD 21205 USA; 9 Department of Physiology, Pharmacology and Therapeutics, Johns Hopkins University School of Medicine, Baltimore, MD 21205 USA; 10 Aligning Science Across Parkinson’s (ASAP) Collaborative Research Network, Chevy Chase, Maryland 20815 USA

**Keywords:** Parkinson’s disease, α-Synuclein, Lewy pathology, Preformed αSyn fibril, Oxidative stress, Retro-AAVs

## Abstract

**Background:**

Parkinson disease (PD) is characterized by progressive neuronal loss within defined brain regions, accompanied by α-synuclein (αSyn)-rich inclusions, termed Lewy pathology (LP). However, it is unclear which cellular factors render certain neuronal populations vulnerable, while others stay devoid of LP throughout the course of disease.

**Methods:**

This study aimed to identify and compare the cellular architecture of vulnerable and non-vulnerable neurons exposed to αSyn pathology by using a projection-based retro-AAV approach in combination with an in vivo α-synucleinopathy mouse model. To do so, a set of viral genetic, immunohistochemical, and optical tools was used in combination with the preformed αSyn fibril (PFF) model.

**Results:**

αSyn pathology propagated robustly into the input connectome of the pedunculopontine nucleus (PPN). However, we observed a marked mismatch between the anatomically expected and the actual distribution of pathology. While anatomically connected neurons in the bed nucleus of the stria terminalis (BST) and the central amygdala (CEA) accumulated substantial αSyn pathology, equally strong connected neurons of the substantia nigra pars reticulata (SNr), and the dentate nucleus (DN) were devoid of pathology. Second, cellular vulnerability and resilience were consistent and reproducible features. When PFFs were injected into alternative major output projection sites of BST, CEA, SNr, and DN, we observed similar patterns of αSyn accumulation. Third, projection-specific axonal mapping revealed that the αSyn-accumulating BST and CEA neurons possessed larger axonal arbors than the more resilient neurons in SNr and DN. Correspondingly, neurons in BST and CEA exhibited higher basal mitochondrial oxidation levels, indicating an increased bioenergetic burden. Finally, the site of initial seeding significantly influenced the extent of developing brain-wide pathology, suggesting that certain brain regions may function as "super-seeders", promoting widespread propagation of pathology, while others contribute relatively little to the global LP burden.

**Conclusions:**

αSyn pathology propagates along anatomical pathways, but cell-autonomous factors determine if a neuron exposed to misfolded αSyn will develop Lewy-like pathology or not.

**Supplementary Information:**

The online version contains supplementary material available at 10.1186/s40035-026-00535-7.

## Background

Clinically, PD is characterized by an akinetic-rigid motor syndrome, which results from the loss of dopaminergic neurons in the substantia nigra pars compacta (SNc) and subsequent dysregulation of the basal ganglia [1, 2]. Histopathologically, neuronal loss is frequently accompanied by α-synuclein (αSyn)-rich inclusions [3–6], termed Lewy pathology (LP). Large postmortem studies have demonstrated that LP is not confined to the SNc, but affects other neuronal populations within the central as well as the peripheral nervous systems, including neurons of the dorsal motor nucleus of the vagus nerve, the pedunculopontine nucleus (PPN), the basal forebrain, and the locus coeruleus (LC) [7–9]. Importantly, not all neurons are equally likely to manifest LP. Previous studies have indicated that LP-bearing neuronal populations often share a common at-risk phenotype. They possess long, unmyelinated axons, are slow autonomous pacemakers with broad action potentials and exhibit low intrinsic calcium-buffering capacity [10–14]. Even within the highly vulnerable SNc, there seems to be a gradient of cellular vulnerability. Transcriptional profiling of human SNc neurons revealed that *AGTR1* (angiotensin II type 1 receptor protein)-positive neurons seem to exhibit the greatest vulnerability to degeneration [15]. Further, in the neurochemically heterogeneous PPN, LP is constrained mostly to the cholinergic subpopulation, sparing GABAergic and glutamatergic neurons [9, 16–18].

Experimental animal studies have shown that stereotaxically injected preformed αSyn fibrils (PFFs) can spread along anatomical pathways, thereby allowing propagation of LP within anatomically connected networks [16, 19–26]. Yet, anatomical connectivity alone is not sufficient to predict which neuronal populations within an affected network will accumulate LP [16], strongly suggesting that cell-autonomous factors determine if a neuron exposed to misfolded αSyn will develop LP or not [27]. Previous experimental studies using αSyn-knockout mice indicated that the presence of endogenous intracellular αSyn plays an important role in promoting templated LP seeding [19]; that is, higher endogenous αSyn expression levels are correlated with the development of LP-like pathology [28–30]. Despite the current progress, several key questions remain unanswered.

A fundamental question is what role cell autonomous factors play in the development of LP, in addition to anatomical connectivity and endogenous αSyn expression levels [27]. To pursue this question, we employed viral, immunohistochemical and optical tools to examine the spread of LP-like pathology following the injection of pre-formed mouse αSyn fibrils into the mouse PPN. The PPN was chosen as the primary injection site, given its relevance in regard to PD motor and non-motor symptoms [31–33], the intrinsic differential vulnerability with cholinergic PPN neurons bearing the brunt of LP and neurodegeneration [16, 18, 34], and the evidence from our previous study [16], that the propagation of αSyn pathology from the PPN is multifactorial and not simply dictated by its anatomical network. This approach not only allowed us to examine the relationship between the extent of propagated pathology and the strength of anatomical connectivity, but also enabled the identification of vulnerable versus resilient neuronal populations. Moreover, it facilitated an analysis of the role of the local seeding site and allowed us to characterize axonal arbor size and mitochondrial oxidative stress levels as key indicators of cellular vulnerability.

## Methods

### Animals

All animal experiments were performed according to the NIH Guide for the Care and Use of Laboratory Animals and approved by the Northwestern University Animal Care and Use Committee. Research was conducted according to the ARRIVE Guidelines. Mice were housed in groups of up to 5 animals per cage with food and water provided ad libitum on a 12-h light/dark cycle. All experiments were performed in the light phase. Throughout the study, wild-type mice (C57BL/6 J; RRID:IMSR_JAX:000664) and in a subset of experiments, heterozygous PV-Cre mice (B6.129P2-Pvalbtm1(cre)Arbr/J; RRID:IMSR_JAX:017320), were used. A similar distribution of male and female mice was used in all experiments. All animals were between two and three months old at the beginning of the experiments.

### Preparation of purified mouse αSyn PFFs and mouse αSyn monomers

Mouse full-length αSyn PFFs were prepared at Johns Hopkins University School of Medicine, Baltimore, USA as previously described [16, 34, 35]. Briefly, to generate αSyn PFFs, purified full-length mouse αSyn monomers were continuously agitated in a thermomixer (Eppendorf) at 1000 rpm and 37 °C for 7 days. Thereafter, the formed αSyn aggregates were sonicated for 30 s at 20% amplitude with a Branson Digital Sonifier (Danbury, CT). Monomeric αSyn and PFFs were frozen at − 80 °C. A subset of the stock solutions was then used for quality control assessments, including structure analysis with transmission electron microscopy (TEM) and in vitro seeding efficiency in primary cortical neurons from C57BL/6 J mice. For TEM analysis, αSyn PFFs were first adsorbed to copper grids (Electron Microscopy Sciences, Hatfield, PA). After washing three times, the grids were negatively stained with uranyl formate. Images were acquired using Philips/FEI BioTwin CM120 Transmission Electron Microscope (Hillsboro, OR). TEM images confirmed the fibrillar morphology of PFFs (~ 100 nm). Next, primary cortical neurons from C57BL/6 J mice were treated with αSyn PFFs. After 7 days of incubation, neurons were fixed with 4% paraformaldehyde (PFA) and permeabilized with 0.2% Triton X100. Then, cells were blocked and incubated with primary anti-αSyn antibody (ab51253, Abcam, Cambridge, MA), confirming the induction of S129-phosphorylated αSyn (p-αSyn) pathology in primary neurons. Last, aliquoted stock solutions were shipped to Northwestern University, Chicago, USA on dry ice and stored at − 80 °C. At Northwestern University, PFFs were thawed, and sterile phosphate-buffered saline (PBS) was added to the solution to achieve a final protein concentration of 2.5 µg/µL. Thereafter, PFFs were sonicated for 90 s at 10% amplitude, aliquoted and finally stored at − 80 °C. On the day of injection, aliquoted PFFs were thawed and briefly vortexed before injection. Protocol: dx.doi.org/10.17504/protocols.io.dm6gpbw28lzp/v1.

### Stereotaxic injections

For all αSyn PFF injections, irrespective of the injected brain region, a total volume of 550 nL PFFs with a concentration of 2.5 µg/µL was injected. In each case the total volume of 550 nL αSyn PFFs was divided and injected into two injection sites to achieve good coverage of the respective brain region [16]. All injections were performed unilaterally into the left hemisphere. For experiments involving retrograde Cre labeling, 100 nL of AAV2-retro-Cre (pENN.AAV.hSyn.Cre.WPRE.hGH, a gift from James M. Wilson; Addgene viral prep # 105553-AAVrg; http://n2t.net/addgene:105553; RRID:Addgene_105553) were injected into the PPN of the left hemisphere. For labeling the axonal projectome and quantification of the axonal arbor size, 50 nL of AAV9-Flex-eGFP (pCAG-FLEX-EGFP-WPRE, a gift from Hongkui Zeng; Addgene viral prep # 51502-AAV9; http://n2t.net/addgene:51502; RRID:Addgene_51502) was injected in the respective brain regions: bed nucleus of the stria terminalis (BST), central amygdala (CEA), substantia nigra pars reticulata (SNr), and dentate nucleus (DN). For mito-roGFP (roGFP targeted to mitochondrial matrix) experiments, 250 nL of AAV9-mito-roGFP (AAV9-CMV-DIO-rev-MTS-roGFP-WPRE, Virovek, lot: 15–373) was injected into BST, CEA, SNr, and DN. The exact stereotaxic coordinates for all injections are provided in Table S1. For the stereotaxic surgery, mice were anesthetized with isoflurane and placed in a stereotaxic frame (David Kopf Instruments, Tujunga, CA) connected with a computer-guided system (Angle Two, Leica Biosystems, Wetzlar, Germany). Next, a glass pipette (P-97 Pipette Puller, Sutter Instruments, Novato, CA), containing the respective viral vectors or αSyn proteins, was navigated to the injection site. After drilling a small hole, the glass pipette was slowly lowered into the brain. Injections were performed at low speed (max. 100 nL/min) with an automated microinjector (IM-300, Narishige, Japan), and the pipette was left for an additional 5 min in the brain after the injection was completed. Protocol: dx.doi.org/10.17504/protocols.io.81wgby191vpk/v1.

### Histology and imaging

Tissue processing and immunohistochemistry was performed as previously described [16, 34, 36]. To achieve a sufficient fixation for further immunohistochemical analysis, mice were anesthetized with a mixture of ketamine (50 mg/kg) and xylazine (4.5 mg/kg) and sacrificed by transcardial perfusion with ice-cold 0.1 M PBS followed by 4% ice-cold PFA for 5 min. After perfusion, mice were decapitated and brains were quickly removed, followed by post-fixation for 3 days in PFA and 3 days in 30% sucrose solution in 0.1 M PBS. Brains were then frozen on dry ice and stored in − 80 °C until sectioning. On the day of sectioning, brains were embedded in tissue freezing media (OCT Compound, Tissue Tek, Sakura Finetek, Torrance, CA) and cut into 30-μm thick consecutive coronal sections using a cryostat microtome (CM3050 S, Leica). All sections spanning the complete rostro-caudal extent of the brain were kept in correct order and stored at 4 °C in cryoprotect-solution (1:1:3 volume ratio of ethylene glycol, glycerol, and 0.1 M PB) until further processing. Immunofluorescence staining used for data analysis or representative images was performed according to the following protocol. Sections were washed 4 × 5 min in 0.1 M PB buffer and blocked for 1 h in 10% normal donkey serum (NDS) in 0.1 M PB with 0.3% Triton X-100 (PBT) at room temperature (RT). Primary antibodies (Table S2) were diluted at the concentration provided in the Table in 10% NDS in PBT and incubated overnight at 4 °C. On the second day, sections were washed 4 × 5 min in PBT, incubated with fluorophore-conjugated, species-specific secondary antibodies for 2 h at RT, and then blocked with 10% NDS in PBT. In most cases, sections were additionally stained with DAPI (Sigma-Aldrich, Darmstadt, Germany, D9542-5MG, 1:10,000 of 5 mg/ml) for 10 min in 0.1 M PB. Before mounting with antifade mounting medium (ProLong Diamond Antifade Mountant, Invitrogen, P36965), sections were washed 5 × 5 min in PBT. Exceptions to this general immunofluorescence staining protocol were made for staining of S129-phosphorylated αSyn, where a streptavidin-based amplification of fluorescence was used. For this, sections were washed 4 × 5 min in 0.1 M PB, and blocked for 1 h in 10% NDS in PBT at RT. The primary antibody (anti-αSyn (pS129), Abcam, ab51253, RRID:AB_869973) was diluted in 10% NDS in PBT and incubated overnight at 4 °C. On the second day, after an initial wash for 4 × 5 min in PBT, sections were incubated with a biotinylated species-specific secondary antibody (biotinylated anti-rabbit, Jackson ImmunoResearch, 711-065-152, 1:1000, RRID:AB_2340593) directed against the rabbit p-αSyn antibody with 10% NDS in PBT for 1 h at RT. Sections were then washed (3 × 5 min in PBT) and incubated with fluorophore-conjugated streptavidin (Streptavidin AlexaFluor647, Jackson ImmunoResearch, 016-600-084, 1:1000) in 10% NDS in PBT for 2 h at RT. Before mounting, sections were additionally stained with DAPI as described above and thereafter washed again 5 × 5 min in PBT. Representative fluorescent images were acquired with a TCS SP8 confocal microscope (Leica) or an AxioImager M2 microscope (Zeiss, Oberkochen, Germany) equipped with an ORCA-Flash 4.0 LT CMOS camera (Hamamatsu Photonics K.K., C11440-42U, Hamamatsu city, Japan). All images were processed with FIJI to enhance signal-to-noise or to rearrange colors of certain image channels. Protocol: dx.doi.org/10.17504/protocols.io.bp2l6xpr1lqe/v1.

### Two photon laser scanning microscopy for ex vivo redox measurements

Mitochondrial oxidant stress was assessed using a redox-sensitive roGFP probe targeted to the mitochondrial matrix, as previously described [34]. Therefore, C57BL/6 J wildtype mice were injected with 100 nL AAV2-retro-hSyn-Cre into the PPN. Four weeks later, 250 nL AAV9-CMV-DIO-rev-MTS-roGFP-WPRE was injected into either CEA, BST, SNr or DN, using the stereotaxic coordinates described in Table S1. As an internal control, we assessed mitochondrial oxidant stress in SNr neurons of heterozygous PV-Cre mice, by injecting 250 nL AAV9-CMV-DIO-rev-MTS-roGFP-WPRE without prior AAV2-retro-hSyn-Cre injection. Fourteen days after injection of the biosensor containing AAV, mice were sacrificed and ex vivo brain slices were prepared. Therefore, mice were anesthetized with a mixture of ketamine (50 mg/kg) and xylazine (4.5 mg/kg) and sacrificed by transcardial perfusion with ice-cold, oxygenated modified artificial cerebrospinal fluid (aCSF) containing in mM: 125 sucrose, 2.5 KCl, 1.25 NaH_2_PO_4_, 25 NaHCO_3_, 0.5 CaCl_2_, 10 MgCl_2_, and 25 glucose. Once perfused, the brain was rapidly removed and 275-μm-thick coronal slices containing the CEA, BST, SNr, and DN region were cut using a vibratome (VT1200S, Leica). Thereafter, brain slices were incubated in oxygenated modified aCSF containing in mM: 135.75 NaCl, 2.5 KCl, 25 NaHCO_3_, 1.25 NaH_2_PO_4_, 2 CaCl_2_, 1 MgCl_2_, and 3.5 glucose at 34 °C for 30 min, then at room temperature for another 30 min before imaging. All solutions were pH 7.4, 310–320 mOsm L^−1^ and continuously bubbled with 95% O_2_/5% CO_2_. Experiments were performed at 32–34 °C. For imaging, slices were transferred to a recording chamber and continuously perfused with modified aCSF at 32–34 °C at a flow rate of 2 mL/min. Fluorescence was measured using an Ultima Laser Scanning Microscope system (Bruker) with a DODT contrast detector to provide bright-field transmission images with an Olympus × 60/0.9 NA lens. A 2P laser (Chameleon Ultra II, Coherent) tuned to 920 nm was used to excite roGFP. Non-de-scanned emission photons were detected with GaAsP photomultiplier tube from 490 to 560 nm. Time series images of the roGFP probe were acquired with 30 frames obtained over ~ 20 s, with 0.197 μm × 0.197 μm pixels and 10 μs dwell time. The dynamic range of the probe was determined with 2 mM dithiothreitol, a reducing agent, and 200 μM aldrithiol, an oxidizing agent, which were used to sequentially perfuse slices. Using this calibration technique, the dynamic range of the expressed fluorophore and the relative probe oxidation can be calculated independently of the expression level of the probe [34]. Time series images were acquired with each to determine the maximal and minimal fluorescence intensity. Time series images were analyzed offline, and fluorescence measurements in multiple regions of interest were evaluated with the background subtracted. Protocol: dx.doi.org/10.17504/protocols.io.x54v9p1e4g3e/v1 (slice preparation). Protocol: dx.doi.org/10.17504/protocols.io.j8nlkomb1v5r/v1 (redox imaging).

### Analysis of input connectivity and p-αSyn pathology in six predetermined brain sections

To systematically compare the retrograde connectivity of the PPN with the extent of propagated p-αSyn pathology, we selected six coronal brain sections (Bregma coordinates: + 0.98, + 0.14, − 1.34, − 3.16, − 5.34, − 6.24 mm) covering the complete rostro-caudal brain axis. These six sections encompassed a total of 129 brain regions, defined according to the Allen Mouse Brain Reference Atlas (RRID:SCR_002978). Anatomical and p-αSyn pathological data were obtained from separate groups of animals to avoid potential confounding interactions between Cre expression and the concurrent presence of p-αSyn pathology. First, the selected sections were stained using immunohistochemical methods targeting either Cre recombinase (anti-Cre Recombinase, AB3120, Merck Millipore, Darmstadt, Germany, 1:1000, RRID:AB_2085748) or p-αSyn (anti-αSyn (pS129), Abcam, ab51253, RRID:AB_869973), as described above. To improve delineation of individual brain regions, all sections were additionally stained with DAPI (1:10,000 of 5 mg/mL, Sigma-Aldrich, Darmstadt, Germany). Next, brain sections were digitized using a Zeiss AxioImager M2 microscope equipped with a 2D slide scanning module (MBF Bioscience, Williston, VT). Thereby, all individual image tiles were automatically stitched together to generate high-resolution composite images. The resulting composite images were then imported into FIJI (RRID:SCR_002285). For each of the six sections, we created a mask using the Allen Reference Atlas (RRID:SCR_002978) and FIJI (RRID:SCR_002285). Each mask included all brain regions present within the corresponding section. To analyze a given section, we first loaded the composite image and applied the corresponding region mask. In cases where the mask did not perfectly match the section due to tissue variability, we manually adjusted the mask to ensure accurate alignment. Next, we performed a background correction by multiplying the background signal by a factor of four before subtracting it from the image. Following this, we quantified the number of Cre- or p-αSyn-positive pixels in each brain region and normalized this value by dividing it by the total number of pixels of the respective brain region. This allowed us to calculate the percentage of pixels positive for Cre recombinase or p-αSyn for each brain region. Fiber tracts and brain ventricles were excluded from the analysis.

### Axonal arbor analysis

To quantify the extent of the axonal projectomes of the BST, CEA, SNr, and DN, we first injected 100 nL of AAV-retro-Cre (RRID:Addgene_105553) into the PPN. Four weeks later, after sufficient Cre expression has taken place, we injected 25 nL of Cre-dependent AAV-Flex-eGFP (RRID:Addgene_51502) into the BST, CEA, SNr, and DN, each in separate cohorts of animals. Two weeks after the second injection, animals were sacrificed, and brains cut into 30 µm-thick coronal sections. To quantify the amount of eGFP-positive axonal projections throughout the complete brain, coronal sections with an interslice distance of 240 µm—covering the full rostro-caudal extent of the mouse brain—were stained for eGFP (anti-eGFP, AB16901, Merck Millipore, 1:1000, RRID:AB_90890) and DAPI (D9542, Sigma-Aldrich, 1:10,000 dilution from 5 mg/mL stock), as described above. Next, sections were imaged using a Zeiss AxioImager M2 microscope equipped with a 2D slide scanning module (MBF Bioscience, Williston, VT), and high-resolution composite images were generated. Using FIJI (RRID:SCR_002285), the left and the right hemispheres were outlined separately within each section, excluding the ventricles. Thereafter, a background correction was performed by multiplying the tissue background signal by a factor of four before subtraction. We then quantified the number of eGFP-positive pixels within each brain section for both hemispheres and normalized it by the total number of pixels, thereby yielding the proportion of eGFP-positive area for each respective brain section. Data from all sections were summarized for the left and the right hemispheres. Because the size of the axonal projectome depends on the number of starter cells, we quantified the starter cell count for each injection site (BST, CEA, SNr, DN). Starter cells were defined by being positive for Cre and eGFP. The axonal arborization data were then normalized to the number of starter cells for BST, CEA, SNr, and DN.

### Whole-brain p-αSyn assessment for different seeding sites

To quantify and compare the brain-wide p-αSyn burden after PFF seeding in CEA, BST, SNc, LC/PB, GRN, VM, SNr, APN, and superior colliculus motor part (SCm), we employed a similar strategy as described for the axonal arbor analysis. Briefly, αSyn PFFs were injected into C57BL/6 J wildtype mice (RRID:IMSR_JAX:000664) using the stereotaxic coordinates described in Table S1. All seeding sites received 550 nL αSyn PFFs from the same batch with a concentration of 2.5 µg/µL. Mice were then sacrificed 12 weeks after stereotaxic surgery and brains processed for immunohistochemical analysis. To quantify brain-wide p-αSyn-positive signal, 30-µm-thick coronal sections with an interslice distance of 240 µm—covering the full rostro-caudal extent of the mouse brain—were stained for p-αSyn (anti-p-αSyn, ab51253, Abcam, 1:2000, RRID:AB_869973) and DAPI (D9542, Sigma-Aldrich, 1:10,000 dilution from 5 mg/mL stock), using the protocol described above. Thereafter, sections were imaged using a Zeiss AxioImager M2 microscope equipped with a 2D slide scanning module (MBF Bioscience), and high-resolution composite images were generated. Using FIJI (RRID:SCR_002285), the left and the right hemispheres were outlined separately within each section, excluding the ventricles. Thereafter, a background correction was performed by multiplying the tissue background signal by a factor of four before subtraction. We then quantified the number of p-αSyn-positive pixels within each coronal brain section for both hemispheres and normalized it by the total number of pixels, thereby yielding the percentage of p-αSyn-positive area for each respective section. Data from all sections were summarized for the left and the right hemispheres for each seeding site.

### Quantitative PCR (q-PCR) of *Snca* expression

To quantify and compare endogenous αSyn expression levels in BST, CEA, SNr, and DN neurons, C57BL/6 J wildtype mice (without prior stereotaxic surgery) were deeply anesthetized with a mixture of ketamine (50 mg/kg) and xylazine (4.5 mg/kg) and sacrificed by transcardial perfusion with ice-cold, oxygenated PBS solution. After perfusion, brains were quickly extracted on ice and 275-μm thick coronal slices containing the CEA, BST, SNr, and DN region were cut using a vibratome (VT1200S Leica Microsystems). The respective brain regions were then precisely dissected using a microscalpel blade. Since we decided to perform the *Snca* PCR using ex vivo fresh brain tissue in order to minimize nucleic acid degradation, the use of laser microdissection was not feasible. However, all four brain regions (BST, CEA, SNr, and DN) could be precisely identified and dissected with appropriate anatomical expertise, ensuring region-specific sampling. Brain tissues containing the respective brain region were then quickly frozen in liquid nitrogen and stored in − 80 °C until further processing. Total RNA from indicated brain tissue sections was then extracted using TRIzol reagent (ThermoFisher Scientific, Darmstadt, Germany) and treated with TURBO DNase (ThermoFisher Scientific) according to manufacturer’s protocol. TURBO Dnase was inactivated by re-extracting RNA with TRIzol reagent. For detection of transcripts, 200 ng of total RNA was reverse transcribed with iScript cDNA synthesis kit (Bio-Rad, Dreieich, Germany) and q-PCR was performed on the StepOnePlus Real-Time PCR System (Applied Biosystems, ThermoFisher Scientific), using iTaq SYBR Green Supermix with ROX (Bio-Rad). Each sample was measured in duplicates. qRT-PCR data were analyzed by the ΔCt method, where Ct values were first normalized to an internal control (*ActB*). The following primers were used for qPCR: *Snca* (F- CCTGGCAGTGAGGCTTATGA, R- AAGGTCATGACTGGGCACAT), *Rp2* (F- CCACAGAAGCCAATAGAAGCA, R- CGGGAGAAGCCTTTACCAAC), *U6* (F- CTCGCTTCGGCAGCACA, R- AACGCTTCACGAATTTGCGT), *Ywhaz* (F- TGGAAGTCCTGCCCTAAATG, R- GAGGAGGAGGAGGAGGAAGA) and *ActB* (F- ATCATTGCTCCTCCTGAGCG, R- ACGCAGCTCAGTAACAGTCC).

### Statistical methods

Data analysis was performed using GraphPad Prism (version 10 GraphPad Software, RRID:SCR_002798), and FIJI (RRID:SCR_002285). In all experiments, sample size was based on prior studies using similar techniques. Sample *n* represents the number of neurons collected from brain slices from *N* animals. Normality of the data was assessed using the Shapiro–Wilk test. When normality was inferred, parametric statistics were performed, otherwise non-parametric testing was conducted. Two-tailed tests were used unless the working hypothesis predicted a clear directionality to the change in outcome measure, in which case one-tailed tests were adopted. Data are presented using box plots showing median values, first and third quartiles, and range, unless otherwise specified. Exact statistical tests are indicated in each figure legend. Differences were considered significant at *P* < 0.05. Reproducibility: all key experiments were independently reproduced by different co-authors. Each experiment was performed multiple times across multiple mice as described in the figure legends. All figures were created with Adobe Illustrator version 25.4 (RRID:SCR_010279).

## Results

### Strength of anatomical connectivity alone does not predict the developing amount of S129-phosphorylated αSyn

**Fig. 1:**
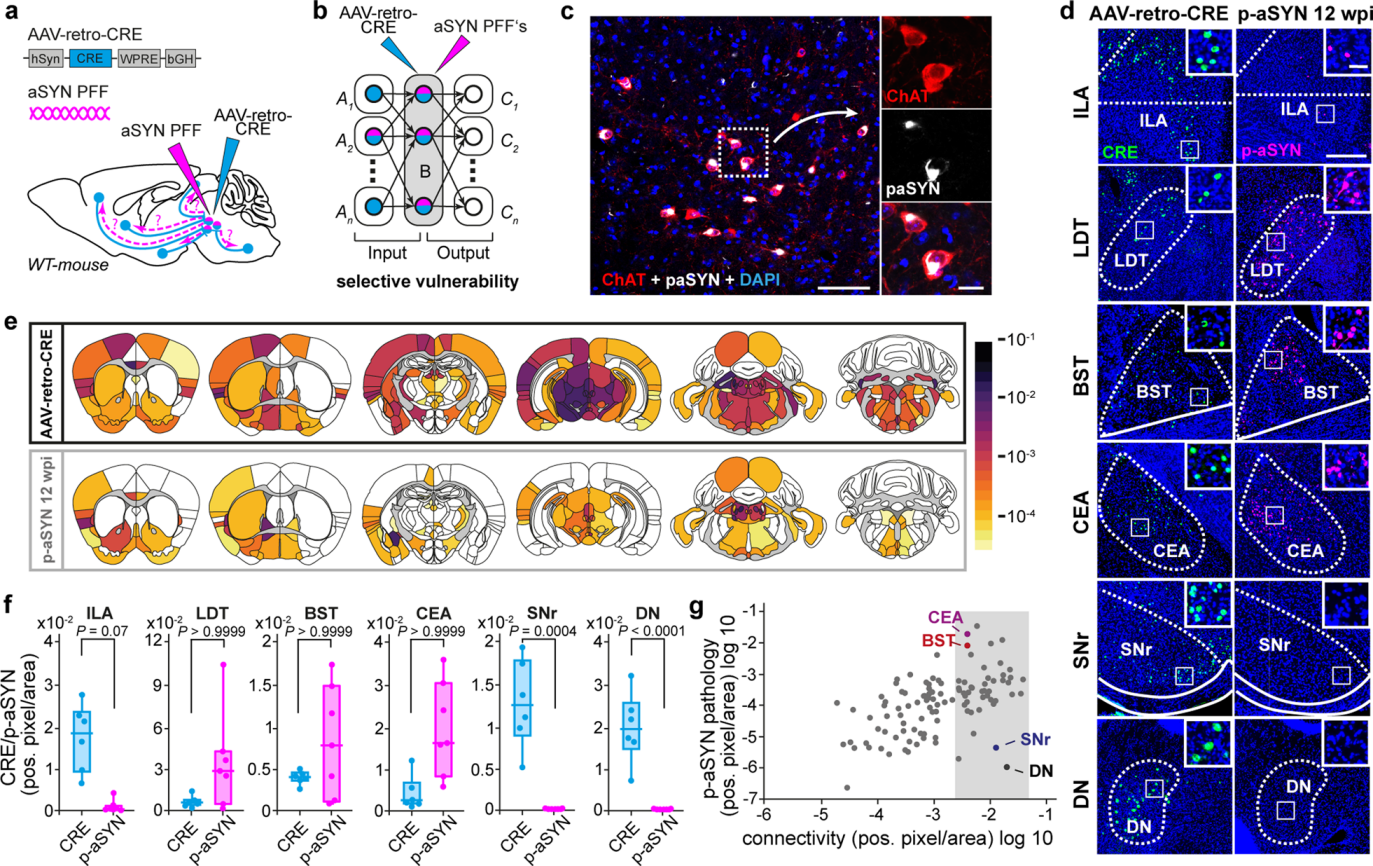
Comparison of input connectivity and PFF pathology spread from the PPN region. **a, b** Schematics showing the strategy of AAV-retro-Cre-based retrograde tracing of PPN neurons and PFF injection into the PPN region of WT mice. **c** PFF-injected PPN region of a WT mouse stained for ChAT (red), p-αSyn (white), and DAPI (blue). Scale bar, 100 μm in overview, 20 μm in high magnification image. **d** Representative images of Cre^+^ input neurons of the PPN (left column) and pathology bearing (p-αSyn^+^) neurons in distinct brain regions. Scale bar, 300 μm in overview, 50 μm in high magnification image. **e** Heatmaps depicting input connectivity strength to the PPN region (upper row) and brain-wide p-αSyn pathology at 12 weeks post-injection (wpi) (lower row). Color scale represents mean positive pixel ratio of Cre (input tracing) and p-αSyn pathology. Quantification includes *N* = 6 for retrograde tracing, and *N* = 7 for p-αSyn pathology. **f** Box plots showing the input strength to the PPN region and p-αSyn pathology in selected brain regions (box plots represent median and interquartile range, whiskers min/max value, CRE *N* = 6, p-αSyn *N* = 7, Kruskal–Wallis test with Dunn’s multiple comparisons). **g** Scatterplots of log p-αSyn pathology versus log input connectivity (Pearson’s* r* = 0.1175, Pearson linear correlation). Abbreviations: BST, bed nucleus of the stria terminalis; CEA, central amygdala; DN, dentate nucleus; ILA, infralimbic area; LDT, laterodorsal tegmental nucleus; SNr, substantia nigra pars reticulata

To investigate the interplay between neuronal connectivity and cell-intrinsic vulnerability, we first stereotaxically injected 550 nL of mouse αSyn-PFFs into the PPN of C57BL/6 J wild-type mice and sacrificed the animals after 12 weeks (Fig. 1a). At this time point, approximately 58% of cholinergic PPN neurons were positive for p-αSyn, indicating robust pathology formation. Consistent with our previous work [16], we observed primarily perinuclear and neuritic p-αSyn-positive inclusions (Fig. 1c and Fig. S1a, b). To assess the brain-wide propagation of p-αSyn pathology in relation to the anatomical input network of the PPN, we injected 100 nL of AAV-retro-CRE into the PPN of a second cohort of C57BL/6 J wild-type mice and sacrificed the animals after 4 weeks. This viral tracing approach enabled us to comprehensively map the input connectome of the PPN and simultaneously provided the basis to further analyze region-specific vulnerability (Fig. 1b and Fig. S2a–c). Next, we quantified both the p-αSyn pathology and CRE recombinase signal in six preselected coronal brain sections, covering the rostro-caudal brain axis, containing a total of 129 brain regions (Fig. 1d, e). Quantification was conducted by determining the number of pixels that were positive for p-αSyn or CRE in a given brain region, followed by normalization to the total area of that region. This approach yielded the percentage of pixels within each region exhibiting p-αSyn or CRE immunoreactivity. This data confirmed that p-αSyn pathology propagated robustly throughout the PPN’s input network. However, several brain regions exhibited a clear mismatch between the extent of p-αSyn pathology and their projection strength to the PPN (Fig. 1e, f). The correlation between the anatomical projection strength and p-αSyn signal across the 129 regions of the ipsilateral hemisphere yielded a Pearson’s *r* of 0.1175. For instance, the CEA and the BST—both strongly connected to the PPN—showed pronounced p-αSyn pathology, whereas the SNr and the DN, which are equally strongly connected, exhibited little to no pathology (Fig. 1f, g).

### Vulnerability and resilience are constant and intrinsic neuronal features

**Fig. 2:**
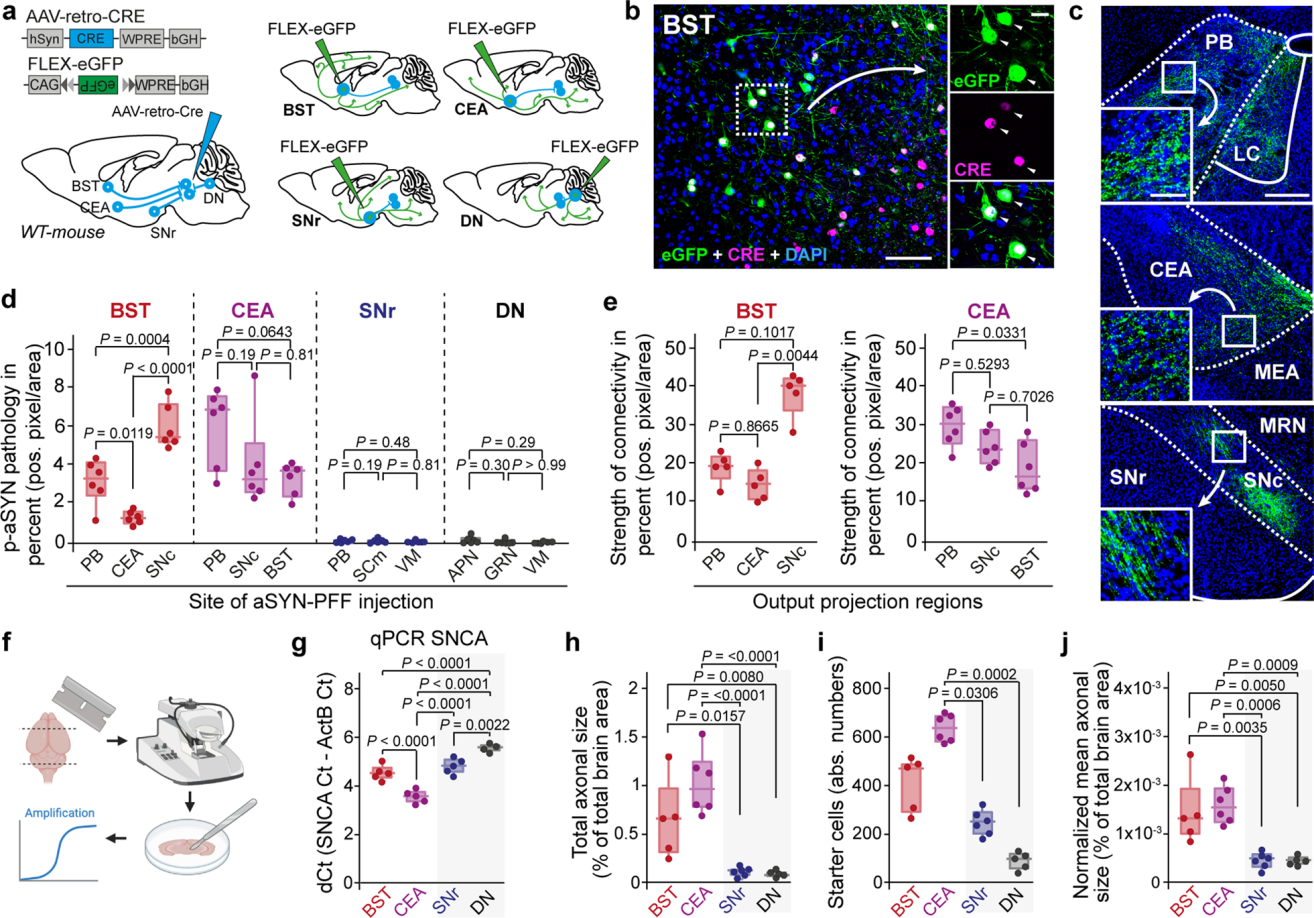
Vulnerability to LP formation is a constant neuronal feature. **a** Experimental protocol. **b** A representative image depicting starter cell population for output tracing of projection-based targeted BST neurons. Neurons staining positive for CRE and eGFP were counted as starter cells (white arrowheads). Scale bar, 100 μm in overview, 25 μm in high magnification image. **c** Representative images depicting three of the major output regions of projection-based targeted BST neurons. Scale bar, 350 μm in overview, 100 μm in high magnification image. **d** Box plots showing propagated p-αSyn pathology in BST, CEA, SNr, and DN following PFF injection in the three major output regions of BST, CEA, SNr, and DN (box plots represent median and interquartile range, whiskers min/max value, *N* = 6 for all groups, for BST, SNr, and DN. Ordinary one-way ANOVA followed by Tukey’s multiple comparisons, for CEA Kruskal–Wallis test with Dunn’s multiple comparisons). **e** Box plots depicting the strength of connectivity between the respective brain regions and BST (left) or CEA (right) (box plots represent median and interquartile range, whiskers min/max value, *N* = 5 for BST injected animals, *N* = 6 for CEA injected animals, Kruskal–Wallis test with Dunn’s multiple comparisons). **f** Scheme for qPCR workflow. **g** *Snca* expression levels in BST, CEA, SNr and DN neurons (box plots represent median and interquartile range, whiskers min/max value, *N* = 5 for BST, CEA, and SNr, *N* = 4 for DN, Ordinary one-way ANOVA followed by Tukey’s multiple comparisons). **h** Total axonal arbor size of projection-based targeted BST, CEA, SNr, and DN neurons (box plots represent median and interquartile range, whiskers min/max value, *N* = 5 for BST, and DN; *N* = 6 for CEA, and SNr, Ordinary one-way ANOVA followed by Tukey’s multiple comparisons). **i** Starter cell counts for the axonal arbor experiment. Starter cells were defined as being positive for CRE and eGFP expression (box plots represent median and interquartile range, whiskers min/max value, *N* = 5 for BST, and DN; *N* = 6 for CEA, and SNr, Ordinary one-way ANOVA followed by Tukey’s multiple comparisons).** j** Normalized mean axonal arbor size of projection-based targeted BST, CEA, SNr, and DN neurons (box plots represent median and interquartile range, whiskers min/max value, *N* = 5 for BST, and DN; *N* = 6 for CEA, and SNr, Ordinary one-way ANOVA followed by Tukey’s multiple comparisons). Abbreviations: APN, anterior pretectal nucleus; BST, bed nucleus of the stria terminalis; CEA, central amygdala; DN, dentate nucleus; GRN, gingatocellular reticular nucleus; LC, locus coeruleus; MEA, medial amygdalar nucleus; MRN, midbrain reticular nucleus; PB, parabrachial nucleus; SCm, superior colliculus, motor part; SNc, substantia nigra pars compacta; SNr, substantia nigra pars reticulata; VM, ventromedial nucleus of the thalamus

Next, we asked whether this differential vulnerability of BST, CEA, SNr, and DN is an intrinsic feature of these neuronal populations, regardless of the primary αSyn seeding site. We hypothesized that injection of mouse αSyn PFFs into other main connectomic partners of BST and CEA should result in significant p-αSyn pathology, whereas injection of mouse αSyn PFFs into partners of SNr and DN should not. To test this hypothesis, we first conducted a projection-specific output tracing to identify the main outputs of BST, CEA, SNr, and DN. Therefore, we injected 100 nL of AAV-retro-CRE into the PPN. Four weeks later, we performed a second stereotaxic surgery and injected 25 nL AAV-Flex-eGFP into BST, CEA, SNr, and DN (Fig. 2a). Two weeks later, we immunohistochemically mapped the output connectomes of BST, CEA, SNr, and DN (Fig. S3). Figure 2b illustrates representative starter cells in BST, while Fig. 2c highlights its three major output projection targets, the parabrachial nucleus (PB), CEA, and SNc. Next, we injected 550 nL of mouse αSyn PFFs into each of these projection targets in different cohorts of C57BL/6 J mice. Twelve weeks later, we quantified the amount of resulting p-αSyn pathology in BST, CEA, SNr, and DN (Fig. 2d). While BST and CEA consistently exhibited pathology, SNr and DN showed little to no pathology, regardless of the initial site of seeding. These data suggest that axonal uptake and retrograde propagation of αSyn fibrils are intrinsic properties of these neuronal populations. Interestingly, even among vulnerable regions, we observed notable variability in the degree of pathology. PFFs injection into the SNc induced significantly more p-αSyn pathology in BST than injection of PFFs into either CEA or PB. Since this approach inherently controls for the intrinsic vulnerability of BST neurons, we hypothesized that this might be due to differences in the anatomical projection strength. Quantitative analysis of the projection strength, defined as the percentage of pixels being positive for axonal eGFP, indeed confirmed that the BST–SNc connection is stronger than those of BST to PB or CEA (Fig. 2e). This data suggest that the strength of anatomical connectivity shapes the extent of propagated αSyn pathology, but only in intrinsically vulnerable neuronal populations.

### Vulnerable neurons share structural and physiological features

Previous studies showed that the presence of endogenous monomeric αSyn is a critical factor for intracellular seeding of αSyn pathology [20]. Therefore, we asked if the lack of pathology in SNr and DN was due to low expression of monomeric αSyn. By utilizing a qPCR approach, we analyzed *Snca* transcript levels in BST, CEA, SNr, and DN (Fig. 2f, g; Fig. S4). Results showed that CEA exhibited the lowest ΔCt values, followed by BST, SNr, and DN—indicating the highest αSyn expression in CEA and the lowest in DN. Importantly, there was no significant difference between BST and SNr (Fig. 2f). Although these results align with previous findings [37], and highlight the role of endogenous αSyn as a cell autonomous vulnerability factor, they do not explain the lack of p-αSyn pathology in SNr and DN.

Several PD-vulnerable regions are part of large neuromodulatory networks, raising the possibility that axonal arbor size is a potential vulnerability factor [27, 38]. Using the AAV-retro-CRE system, we aimed to quantitatively assess the size of the axonal projectome of mouse αSyn PFF-exposed neurons in BST, CEA, SNr, and DN. To do so, we injected 100 nL of AAV-retro-CRE into the PPN, followed four weeks later by 25 nL AAV-Flex-eGFP. Two weeks after the second injection, mice were sacrificed and the axonal projectome of BST, CEA, SNr and DN neurons assessed by quantification of the percentage of pixels being positive for axonal eGFP in a brain-wide manner from the olfactory bulb to the caudal medulla. Results showed that BST and CEA neurons exhibited significantly larger axonal arbors than SNr and DN neurons (Fig. 2h, j). By normalizing the total axonal signal to the number of starter cells in each region, we confirmed that BST and CEA neurons indeed possess a larger axonal arbor than SNr and DN neurons (Fig. 2i, j).

**Fig. 3:**
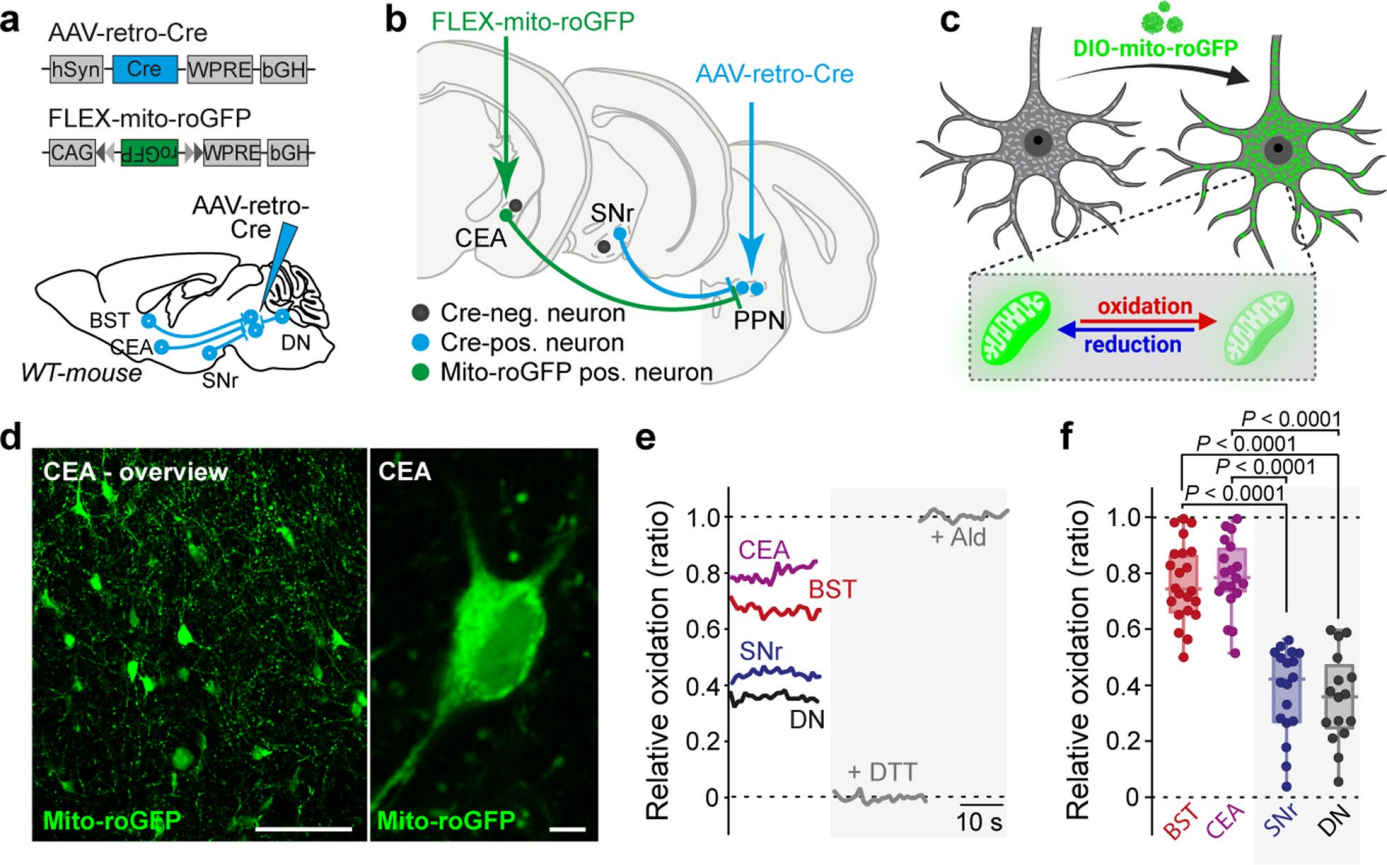
Basal mitochondrial oxidative stress is elevated in vulnerable neurons. **a, b** Experimental protocol.** c** Cartoon depicting characteristics of the optical biosensor. Following delivery of mito-roGFP expressing probe, roGFP is expressed in mitochondria of CRE-expressing neurons. RoGFP is a redox sensitive probe as the emitted fluorescence intensity is dependent on the oxidative state of the probe. **d** Representative images of mito-roGFP-expressing CEA neurons. Scale bar, 250 μm in overview, 10 μm in high magnification image. **e** Calibration protocol of the mito-roGFP probe. **f** Basal mitochondrial oxidative stress levels are higher in vulnerable neurons (BST, CEA) compared to resistant neurons (SNr, DN) (box plots represent median and interquartile range, whiskers min/max value; BST (*N* = 5, *n* = 22), CEA (*N* = 5, *n* = 19), SNr (*N* = 5, *n* = 18), DN (*N* = 5, *n* = 16), Kruskal–Wallis test with Dunn’s multiple comparisons)

A large axonal arbor could increase the vulnerability to αSyn pathology in several ways. One possibility is that it could create a higher bioenergetic demand, which could lead to an intracellular environment that was conducive to LP [39]. To quantify the basal mitochondrial oxidation of BST, CEA, SNr, and DN neurons, 100 nL of AAV-retro-CRE was injected into the PPN of C57BL/6J mice, followed four weeks later by a second stereotaxic injection of 100 nL of a CRE-dependent genetically encoded mitochondrial redox sensor (AAV-DIO-mito-roGFP) into BST, CEA, SNr, and DN. Two weeks later, mitochondrial oxidative stress was assessed in acute brain slices via two-photon laser scanning microscopy (Fig. 3a, b) [34]. Based on the level of mitochondrial oxidation, the emission profile of the mito-roGFP-sensor differs, which can be quantified and translated into the relative oxidation ratio (Fig. 3c–e). These experiments revealed that BST and CEA neurons display a significantly higher level of mitochondrial oxidative stress than SNr and DN neurons (Fig. 3e, f). To rule out interfering effects from the retro-AAV approach, we compared SNr mitochondrial oxidation levels using the AAV-retro-CRE approach with those measured in mice expressing CRE in parvalbumin-positive SNr neurons, which revealed similar results (Fig. S5a–c).

### Distinct brain regions might act as “super-seeders” of brain-wide pathology

**Fig. 4:**
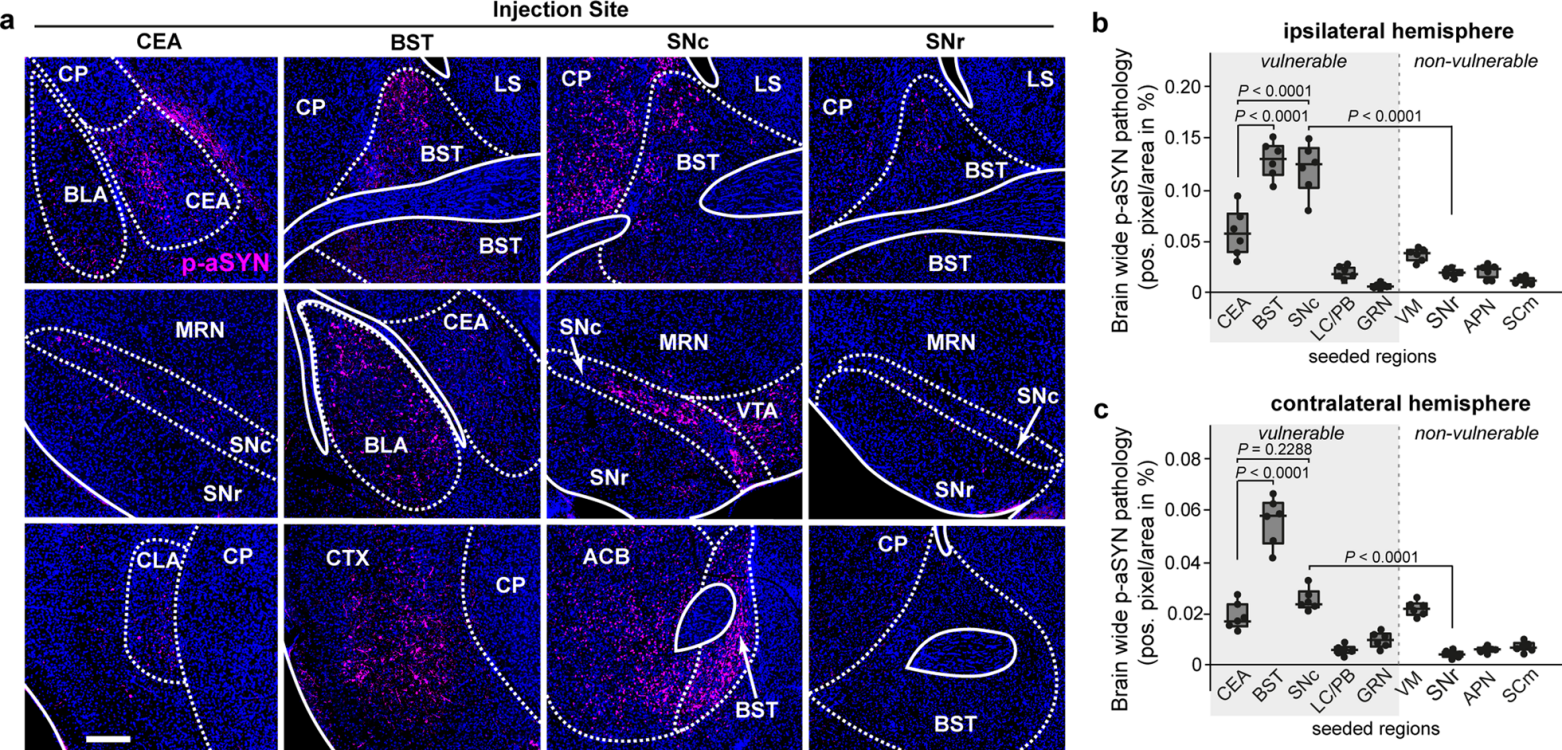
Initial seeding site strongly influences the degree of developing brain-wide p-αSyn pathology. **a** Representative images of brain-wide p-αSyn pathology at 12 wpi following PFF-mediated αSyn seeding in different brain regions. Scale bar, 250 μm. **b, c** Quantification of the burdens of brain-wide p-αSyn pathology in the ipsilateral (**b**) and contralateral (**c**) hemispheres, following injection of equal amounts of αSyn PFFs into nine different brain regions. APN, anterior pretectal nucleus; BST, bed nucleus of the stria terminalis; CEA, central amygdala; GRN, gingatocellular reticular nucleus; LC, locus coeruleus; MRN, midbrain reticular nucleus; PB, parabrachial nucleus; SCm, superior colliculus, motor part; SNc, substantia nigra pars compacta; SNr, substantia nigra pars reticulata; VM, ventromedial nucleus of the thalamus

Finally, we asked whether the site of initial αSyn seeding might influence the amount of brain-wide pathology and could thereby serve as a cell-extrinsic factor of pathology development. To address this question, we injected equal amounts of mouse αSyn PFFs (550 nL with a concentration of 2.5 µg/µL) from the same PFF batch into nine different brain regions (CEA, BST, SNc, LC/PB, nucleus gigantocellularis (GRN), ventral medial nucleus of the thalamus (VM), SNr, anterior pretectal nucleus (APN), and SCm), and systematically quantified the global burden of p-αSyn pathology separately for the ipsilateral and contralateral hemispheres. We observed striking differences in the extent of brain-wide p-αSyn pathology in relation to the primary site of PFF injection. While injection of mouse αSyn PFFs into the CEA, BST, and SNc induced robust pathology within the ipsilateral hemisphere, injections into the LC/PB and GRN, which are also considered vulnerable in PD, led to significantly less brain-wide pathology (Fig. 4a, b, Table S3). Similar results were observed for the contralateral hemisphere. Notably, injection of mouse αSyn PFFs into the BST led to particularly extensive pathology in both hemispheres (Fig. 4b, c, Table S4). In contrast, injection of the same amount of PFFs into the non-vulnerable regions VM, SNr, APN, and SCm resulted in significantly less brain-wide pathology. Most strikingly, injection of mouse αSyn PFFs into the SNr, placed less than a millimeter below the SNc injection coordinates, resulted in only minimal brain-wide pathology. Surprisingly, injection of PFFs into the LC did not induce the formation of p-αSyn pathology within LC neurons and resulted in only mild brain-wide pathology. The lack of p-αSyn seeding within LC neurons was also observed in mice in which mouse αSyn-PFFs were injected into main LC output projection areas (Fig. S6).

## Discussion

There are three main conclusions to be drawn from these studies. First, both anatomical connectivity and cell-autonomous factors govern the propagation of αSyn pathology. Second, neurons that were more vulnerable to propagated αSyn pathology had larger axonal arbors and higher basal levels of mitochondrial oxidant stress. Third, the site of initial αSyn seeding strongly influenced the spread of αSyn pathology.

### Axonal arbor size and mitochondrial stress as cell-intrinsic vulnerability factors

Previous studies have indicated that anatomical connectivity in combination with the level of endogenous αSyn expression is the primary determinant of the propagated p-αSyn pathology in the brain [16, 20, 23, 40, 41]. While our experiments largely align with this body of work, they also demonstrate the limitations of these factors and identify new ones. The fact that p-αSyn pathology was absent in SNr and DN neurons despite the expression of endogenous αSyn, indicates that, at least in some neuronal populations, anatomical connectivity together with endogenous αSyn is not sufficient for LP induction, highlighting the importance of additional vulnerability factors. Identifying those cell-autonomous factors that gate vulnerability [38] is a key task, especially in context of the effort to develop disease-modifying therapies [42]. In that regard, cellular bioenergetics and mitochondrial dysfunction are important targets [34, 39]. Our experiments revealed that vulnerable neurons in the CEA and BST possess significantly larger axonal arbors, along with higher levels of mitochondrial oxidative stress. This data are thereby in line with previous histopathological studies [43] which identified neurons with long unmyelinated axons as predominantly affected by αSyn pathology. We suggest that there is a direct relationship between axonal arbor size, elevated bioenergetic demand, and neuronal vulnerability to αSyn pathology. This hypothesis is further supported by genetic evidence from large-scale PD genome-wide association studies. Several of the most consistently replicated PD risk loci encode proteins involved in mitochondrial quality control, oxidative stress handling, or broader metabolic regulation (e.g., *PINK1*, *PRKN*, *DJ-1*, *LRRK2*, and genes regulating mitochondrial ribosome or autophagy pathways) [44–47]. Cell-type specific genetic profiling further linked mitochondrial dysfunction to αSyn vulnerability [48]. Taken together, these findings support the view that impaired bioenergetics and vulnerability to oxidative stress are not only cellular correlates but also mechanistically relevant contributors to PD pathogenesis.

Large scale cell-type specific profiling of αSyn pathology-bearing neurons [49] or those at risk [15, 48, 50] has identified additional possible vulnerability factors, which need to be tested in experimental models. It is worth noting that these cell autonomous factors might not be the same for all neuronal populations [51]. For example, the lack of αSyn seeding in LC neurons – despite their large axonal arbor and elevated mitochondrial oxidant stress [13] – further suggests that there might be other factors that need to be taken into account [52].

### The site of initial αSyn seeding shapes the brain-wide pathology burden

Another important and previously underappreciated factor is the role of the primary seeding site, where αSyn pathology is initiated [19, 41, 53–55]. Our data clearly show that αSyn seeding with mouse αSyn PFFs in SNc, BST, and CEA led to significantly more widespread brain pathology compared to seeding in other regions (e.g., GRN, SNr, and APN), suggesting that certain brain regions may act as “super-seeders” that promote widespread propagation, while others contribute relatively little to the overall pathological burden. It might be that those “super-seeder” regions are simply anatomical highways with an extensive neuronal network [40, 41]. However, it is also conceivable that the intracellular environment of neurons in these regions potentially modulates the conformation of the αSyn species after cellular uptake [56], raising the possibility that those regions generate toxic αSyn species with increased ability to propagate. Thus, these regions might accelerate the overall disease progression.

Notably, BST seeding resulted in pronounced pathology in the piriform and entorhinal cortices – areas commonly affected in dementia with Lewy bodies [57–59]. From a clinical perspective, this raises the possibility that patients with early BST involvement may be at greater risk of developing cognitive decline. Further, seeding αSyn pathology in the BST might allow modeling dementia with Lewy bodies and thereby offer an opportunity to investigate the vulnerability of affected cortical and hippocampal neurons, while simultaneously providing a platform to study cognitive impairments.

### Relevance of different αSyn strains

One important caveat to consider when translating our results into the human context is that not one single αSyn species exists, but rather a wide range of oligomeric and fibrillar strains, each with distinct structures and biological activities [56, 60–62]. Electron microscopy studies have revealed major structural differences between mouse αSyn PFFs, human αSyn PFFs, as well as human Lewy body-derived versus human MSA-derived fibrils [63–65]. In this study, we chose to perform our experiments with highly standardized recombinant mouse αSyn PFFs [16, 34, 66]. In contrast to human αSyn PFFs, mouse PFFs reliably induce robust aggregation and transmission of pathology in non-transgenic mice, making them a widely used in vivo synucleinopathy model. Would have the use of human PFFs [64] or amplified human Lewy body-derived αSyn [67] led to a different pathology propagation pattern? Based on the structural differences mentioned above, this is conceivable [67, 68], though it would need to be tested experimentally. In this regard, the closest approximation to the human condition might be the use of human αSyn PFFs or amplified human Lewy body-derived αSyn in a humanized animal (e.g. mouse) model. With regard to our findings, such an approach could, for example, help determine whether the lack of vulnerability observed in the LC is strain-dependent. While our mouse-PFF-based experiments produced a propagation pattern that aligns well with human postmortem data [7, 8], using a different αSyn strain might have yielded a different pathology propagation pattern.

Another important modulator of αSyn pathology propagation and toxicity appears to be its protein conformation. During the aggregation process – from monomeric αSyn to insoluble Lewy bodies – monomers first convert to oligomers, then protofibrils, and finally mature fibrils [69]. Moreover, fibrillar αSyn can also release smaller oligomeric species [70]. These different αSyn species, varying in size, structure, and morphology, coexist intracellularly and appear to be in a dynamic equilibrium. Recent studies suggested that oligomeric αSyn species could even be more toxic than the fibrillar forms [70, 71]. In addition, there is also evidence that, in PD patients, the amount of αSyn oligomers in a given brain region does not correlate with the amount of LP present. For example, the occipital cortex – generally devoid of LP – exhibited notable amounts of αSyn oligomers [72]. Therefore, although technically challenging due to the metastability of αSyn oligomers [73], it would be highly informative to compare the propagation pattern we observed using mouse αSyn PFFs with that induced by αSyn oligomers [74].

### Potential role of the microglial environment

While we focused on cell-autonomous neuronal vulnerability factors, the microglial environment likely also plays a crucial role in the cell-to-cell transmission of αSyn pathology. Microglia take up monomeric, oligomeric, and fibrillar forms of αSyn and degrade them via autophagy, entering an activated state in the process [75]. Impairment of microglial autophagy has been associated with enhanced neurodegeneration [76]. Notably, microglial cells show regional heterogeneity in their gene expression patterns, functions and metabolism [77, 78]. This heterogeneity might contribute to the observed regional differences of pathology initiation and progression. However, microglia heterogeneity does not explain the cell-type-specific vulnerability of certain neuronal subpopulations within the same region. The PPN illustrates this well. Despite a shared microglial environment, cholinergic neurons almost exclusively acquire αSyn pathology while glutamatergic and GABAergic PPN neurons remain largely unaffected [16, 34].

## Conclusion

Within this study, we identified and compared the cellular architecture of vulnerable and non-vulnerable neurons exposed to propagated αSyn pathology. We provide experimental evidence that axonal arbor size and mitochondrial stress are associated with increased cellular vulnerability to αSyn pathology formation. Moreover, the observation that the site of initial αSyn seeding strongly influenced the extent of propagated αSyn pathology, suggests that certain brain regions may function as "super-seeders", while others contribute relatively little to the global pathology burden.

## Supplementary Information

Additional file 1. **Figure S1**. PFF injection into the PPN region leads to p-αSYN pathology in cholinergic PPN neurons. **Figure S2**. AAV-retro-CRE based input tracing of PPN neurons. **Figure S3**. Output tracing of projection-based targeted BST, CEA, SNr, and DN neurons. **Figure S4**. Control qRT-PCR measurements of housekeeping genes in BST, CEA, SNr, and DN. **Figure S5**. Mitochondrial oxidative stress measurements of SNr neurons. **Figure S6**. Seeding deficiency of PFFs in noradrenergic LC neurons. **Table S1**. Coordinates of stereotactic surgery of respective brain regions. **Table S2**. Primary and secondary antibodies and the dilutions used in the study. **Table S3**. *P* values global pathology on contralateral hemisphere. **Table S4**. *P* values global pathology on ipsilateral hemisphere.

## Data Availability

The datasets generated and/or analyzed during the current study are available in the Zenodo repository (https://doi.org/10.5281/zenodo.16601256).

## Abbreviations

aCSF: Artificial cerebrospinal fluid
APN: Anterior pretectal nucleus
αSyn: Alpha-synuclein
BST: Bed nucleus of the stria terminalis
CEA: Central amygdala
ChAT: Choline acetyltransferase
CRE: CRE recombinase
DIO: Double-floxed inverse open reading frame
GABA: Gamma-aminobutyric acid
GRN: Nucleus gigantocellularis
LC: Locus coeruleus
PB: Parabrachial nucleus
PBS: Phosphate-buffered saline
PD: Parkinson’s disease
PFA: Paraformaldehyde
PFFs: Pre-formed alpha-synuclein fibrils
PPN: Pedunculopontine nucleus
p-αSyn: S129-phosphorylated alpha-synuclein
eGFP: Enhanced green fluorescent protein
roGFP: Redox-sensitive variant of green fluorescent protein
SCm: Superior colliculus, motor part
SNc: Substantia nigra pars compacta
SNr: Substantia nigra pars reticulata
TEM: Transmission electron microscopy
VM: Ventral medial nucleus of the thalamus
