# Supplementary material for: Mapping cellular vulnerability in Parkinson’s disease using retro-AAVs and preformed α-synuclein fibrils
Source: Transl Neurodegener. 2026 Jan 30;15:2. doi: 10.1186/s40035-026-00535-7 (PMC12859986; doi:10.1186/s40035-026-00535-7)
Supplement: Supplementary file 1 — Additional file 1. Figure S1. PFF injection into the PPN region leads to p-αSYN pathology in cholinergic PPN neurons. Figure S2. AAV-retro-CRE based input tracing of PPN neurons. Figure S3. Output tracing of projection-based targeted BST, CEA, SNr, and DN neurons. Figure S4. Control qRT-PCR measurements of housekeeping genes in BST, CEA, SNr, and DN. Figure S5. Mitochondrial oxidative stress measurements of SNr neurons. Figure S6. Seeding deficiency of PFFs in noradrenergic LC neurons. Table S1. Coordinates of stereotactic surgery of respective brain regions. Table S2. Primary and secondary antibodies and the dilutions used in the study. Table S3. P values global pathology on contralateral hemisphere. Table S4. P values global pathology on ipsilateral hemisphere. [file 40035_2026_535_MOESM1_ESM.pdf]

Supplementary Materials for

Mapping cellular vulnerability in Parkinson's disease using retro-AAVs and preformed α-synuclein fibrils

Fanni F. Geibl, Ahmed A. Musa, Leo Dietrich, Helena Wolter, David L. Wokosin, Sharof Khudayberdiev, Marco B. Rust, Rong Chen, Valina L. Dawson, Ted M. Dawson, Wolfgang H. Oertel, D. James Surmeier, Martin T. Henrich[§]

[§]Corresponding author. E-mail: martin.henrich@uni-marburg.de

This PDF file includes:

Figs. S1 to S6

Supplementary Tables 1 to 4

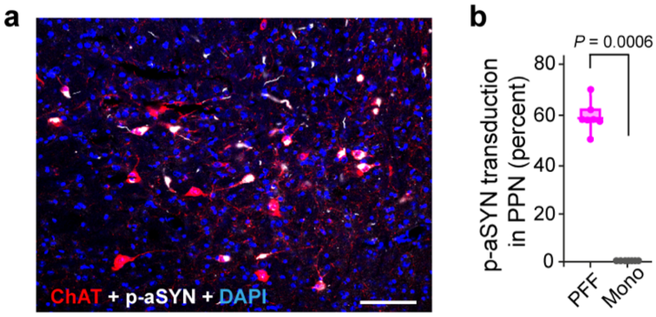

Supplementary Figure 1. PFF injection into the PPN region leads to p- αSyn pathology in cholinergic PPN neurons. a Representative image depicting p-αSyn pathology (white) in $ChAT^+$ (red) PPN neurons. Scale bar, 100 μm. **b** Box plot showing transduction rates of cholinergic PPN neurons of PFF injected mice versus monomeric αSyn injected mice (box plots represent median and interquartile range, whiskers min/max value; *N*=7 for both groups, Mann-Whitney U test).

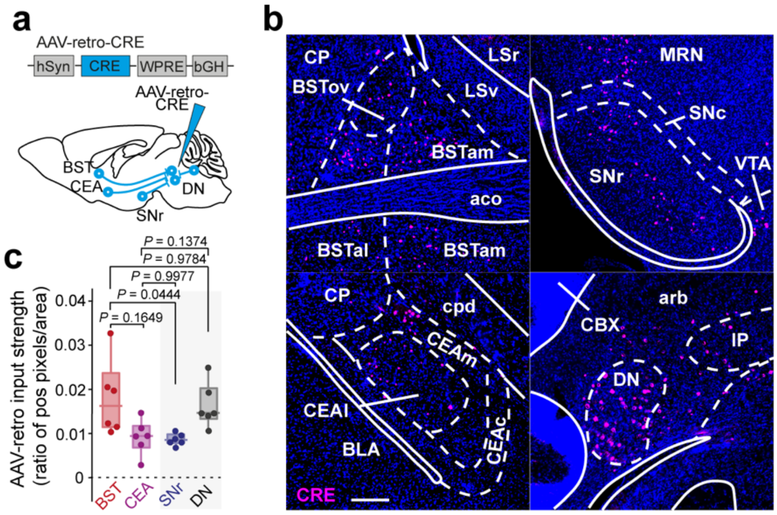

Supplementary Figure 2. AAV-retro-CRE based input tracing of PPN neurons. a Experimental protocol. **b** Representative images of CRE-expressing input neurons in four selected brain regions. Scale bar, 200 µm. **c** Input strength of the four selected brain regions (box plots represent median and interquartile range, whiskers min/max value; *N*=6, Kruskal-Wallis test with Dunn's multiple comparisons). Abbreviations: aco, anterior commissure; arb, arbor vitae; BLA, basolateral amygdala; BSTal, bed nucleus of the stria terminalis, anterolateral area; BSTam, bed nucleus of the stria terminalis, anteromedial area; BSTov, bed nucleus of the stria terminalis, oval nucleus; CBX, cerebellum; CEAc, central amygdala, capsular part; CEAl, central amygdala, lateral part; CEAm, central amygdala, medial part; CP, caudoputamen; cpd, cerebral peduncule; DN, dentate nucleus; IP, interpeduncular nucleus; LSr, lateral septal nucleus, rostral part; LSv, lateral septal nucleus, ventral part; MRN, midbrain reticular nucleus; SNc, substantia nigra pars compacta; SNr, substantia nigra pars reticulata; VTA, ventral tegmental area.

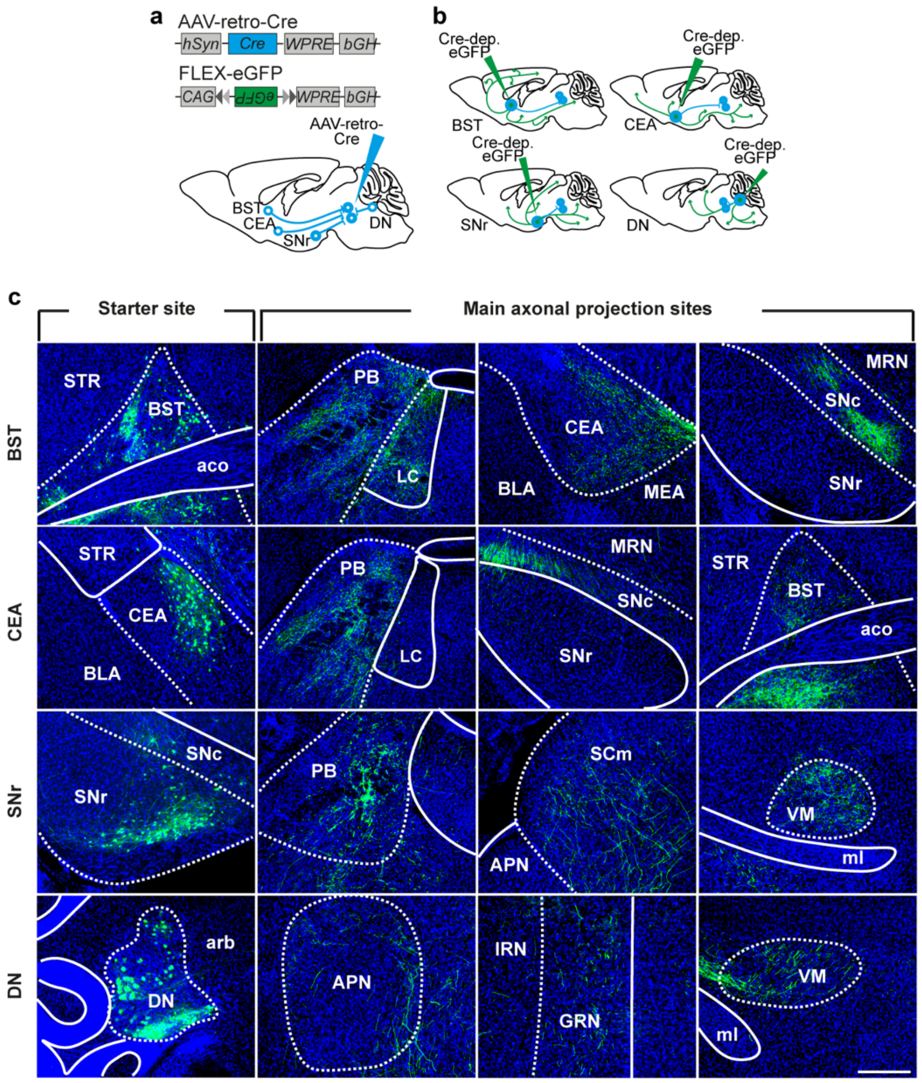

Supplementary Figure 3. Output tracing of projection-based targeted BST, CEA, SNr, and DN neurons. a, b Experimental protocol. **c** Representative images of the starter cells and three major output regions of the respective brain regions. Scale bar, 500 µm. Abbreviations: aco, anterior commissure; APN, anterior pretectal nucleus; arb, arbor vitae; BLA, basolateral amygdala; BST, bed nucleus of the stria temrinalis; CEA, central amygdala; DN, dentate nucleus; GRN, gigantocellular reticular nucleus; IRN, intermediate reticular nucleus; LC, locus coeruleus; MEA, medial amygdalar nucleus; ml, medial lemniscus; MRN, midbrain reticular nucleus; PB, parabrachial nucleus; SCm, superior colliculus, motor part; SNc, substantia nigra pars compacta; SNr, substantia nigra pars reticulata; STR, striatum; VM, ventromedial nucleus of the thalamus.

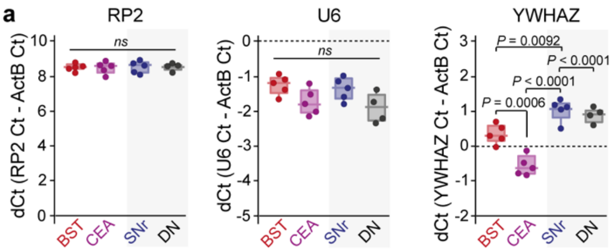

Supplementary Figure 4. Control qRT-PCR measurements of housekeeping genes in BST, CEA, SNr, and DN. a qRT-PCR measurements of the housekeeping genes RP2, U6, YWHAZ (box plots represent median and interquartile range, whiskers min/max value; N=5 for BST, CEA, and SNr; N=4 for DN, Kruskal-Wallis test with Dunn's multiple comparisons).

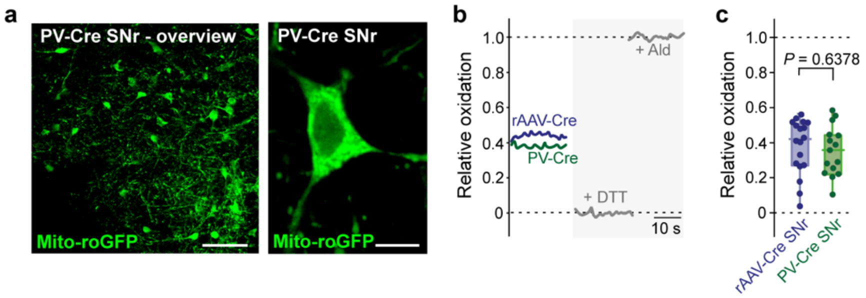

Supplementary Figure 5. Mitochondrial oxidative stress measurements of SNr neurons. **a** Representative image of mito-roGFP expressing PV^{+} SNr neurons in PV-Cre mice. Scale bar, 500 μm in overview, 20 μm in high magnification image. **b** Calibration protocol. **c** No significant difference between basal mitochondrial oxidative stress of PV^{+} SNr neurons in PV-Cre animals compared to projection-based targeted SNr neurons (box plots represent median and interquartile range, whiskers min/max value; rAAV-Cre SNr (*N*=5, *n*=18); PV-Cre SNr (*N*=5, *n*=15), Mann-Whitney U test).

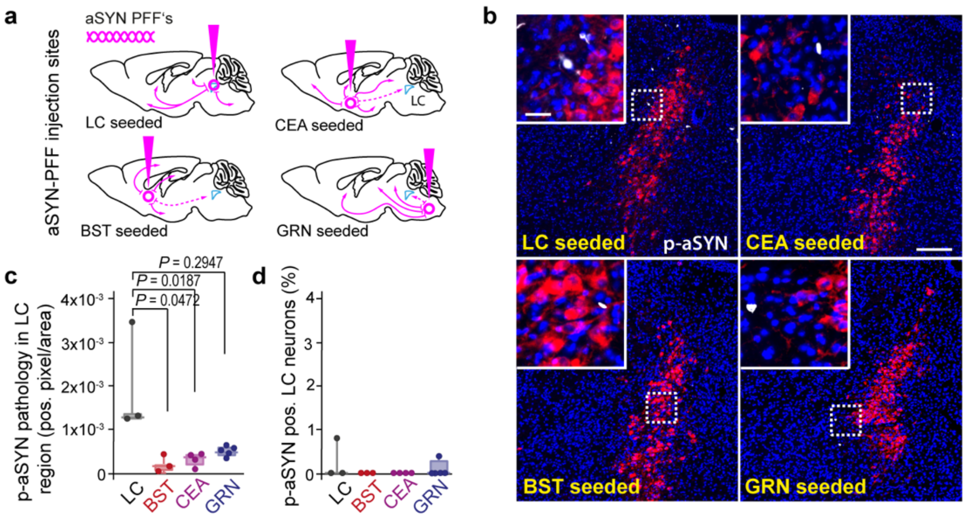

Supplementary Figure 6. Seeding deficiency of PFFs in noradrenergic LC neurons. a Experimental protocol. **b** Representative images of TH^+ noradrenergic LC neurons following PFF injection into the LC region directly or three major LC output regions (CEA, BST, GRN). Scale bar, 300 μm in overview, 50 μm in high magnification images. **c** Box plots showing p-αSyn positive pathology in the LC region of mice injected with aSYN PFF into LC, BST, CEA, or GRN (box plots represent median and interquartile range, whiskers min/max value; *N*=3 for LC and BST, *N*=4 for CEA, *N*=5 for GRN seeded mice; Kruskal-Wallis test with Dunn's multiple comparisons). **d** Graph showing transduction rate of TH^+ noradrenergic LC neurons of mice injected with aSYN PFF into LC, BST, CEA, or GRN (box plots represent median and interquartile range, whiskers min/max value; *N*=3 for LC and BST, *N*=4 for CEA, *N*=5 for GRN seeded mice).

	1. injection spot			2. injection spot		
	ML (mm)	AP (mm)	DV (mm)	ML (mm)	AP (mm)	DV (mm)
**PPN**	-1.23	-4.60	-3.56	-1.10	-4.36	-3.70
**SNr**	-1.50	-3.52	-4.45	-1.40	-3.10	-4.50
**CEA**	-2.40	-1.58	-4.40	-2.35	-1.20	-4.60
**VM**	-0.70	-1.78	-4.20	-0.85	-1.44	-4.20
**BST**	-0.85	+0.18	-4.80	-0.70	+0.36	-4.70
**SCm**	-1.20	-3.45	-2.45	-1.30	-3.85	-2.25
**SNc**	-1.30	-3.50	-4.20	-1.30	-3.10	-4.45
**DN**	-2.25	-6.00	-3.74			
**LC/PB**	-1.05	-5.40	-3.65	-1.00	-5.65	-3.75
**APN**	-1.35	-3.20	-3.20	-1.25	-2.90	-2.95
**GRN**	-0.30	-5.80	-5.25	-0.50	-6.36	-5.50

Supplementary Table 1. Coordinates of stereotactic surgery of respective brain regions

	Host	Cat. No.	Manufacturer	Dilution	RRID
**Primary antibodies**					
Cre recombinase	Mouse	MAB3120	Merck Millipore	1:1000	AB_2085748
Choline Acetyltransferase (ChAT)	Goat	AB144P	Merck Millipore	1:100	AB_2079751
Tyrosine Hydroxylase (TH)	Chicken	AB9702	Sigma Aldrich	1:1000	AB_570923
Alpha-synuclein (p-S129)	Rabbit	ab51253	Abcam	1:2000	AB_869973
Green fluorescent protein (GFP)	Chicken	AB16901	Merck Millipore	1:1000	AB_90890
**Secondary antibodies**					
Anti-chicken AlexaFluor488	Donkey	703-545-155	Jackson ImmunoResearch	1:1000	AB_2340375
Anti-chicken Cy3	Donkey	703-165-155	Jackson ImmunoResearch	1:1000	AB_2340363
Anti-goat Cy3	Donkey	705-165-147	Jackson ImmunoResearch	1:1000	AB_2307351
Anti-mouse AlexaFluor488	Donkey	A-21202	Invitrogen	1:1000	AB_141607
Biotinylated anti-rabbit	Donkey	711-065-152	Jackson ImmunoResearch	1:1000	AB_2340593
Biotinylated anti-mouse	Donkey	715-065-151	Jackson ImmunoResearch	1:1000	AB_2340785
Streptavidin AlexaFluor647	Donkey	016-600-084	Jackson ImmunoResearch	1:1000	AB_2341101

Supplementary Table 2. Primary and secondary antibodies and the dilutions used in the study.

Comparison	Mean Diff.	95% CI of diff.	Adjusted P value
CEA vs. BST	-0,03723	-0,04516 to -0,02931	<0,0001
CEA vs. SNc	-0,006249	-0,01418 to 0,001677	0,2288
CEA vs. LC/PB	0,01268	0,004757 to 0,02061	0,0001
CEA vs. GRN	0,009274	0,001348 to 0,01720	0,0114
CEA vs. VM	-0,003119	-0,01105 to 0,004807	0,9315
CEA vs. SNr	0,01476	0,006836 to 0,02269	<0,0001
CEA vs. APN	0,01275	0,004824 to 0,02068	0,0001
CEA vs. SCm	0,01229	0,004363 to 0,02021	0,0002
BST vs. SNc	0,03099	0,02306 to 0,03891	<0,0001
BST vs. LC/PB	0,04992	0,04199 to 0,05784	<0,0001
BST vs. GRN	0,04651	0,03858 to 0,05443	<0,0001
BST vs. VM	0,03412	0,02619 to 0,04204	<0,0001
BST vs. SNr	0,05200	0,04407 to 0,05992	<0,0001
BST vs. APN	0,04999	0,04206 to 0,05791	<0,0001
BST vs. SCm	0,04952	0,04160 to 0,05745	<0,0001
SNc vs. LC/PB	0,01893	0,01101 to 0,02686	<0,0001
SNc vs. GRN	0,01552	0,007597 to 0,02345	<0,0001
SNc vs. VM	0,003130	-0,004796 to 0,01106	0,9302
SNc vs. SNr	0,02101	0,01308 to 0,02894	<0,0001
SNc vs. APN	0,01900	0,01107 to 0,02693	<0,0001
SNc vs. SCm	0,01854	0,01061 to 0,02646	<0,0001
LC/PB vs. GRN	-0,003410	-0,01134 to 0,004516	0,8915
LC/PB vs. VM	-0,01580	-0,02373 to -0,007877	<0,0001
LC/PB vs. SNr	0,002078	-0,005848 to 0,01000	0,9943
LC/PB vs. APN	6,698e-005	-0,007859 to 0,007993	>0,9999
LC/PB vs. SCm	-0,0003944	-0,008320 to 0,007532	>0,9999
GRN vs. VM	-0,01239	-0,02032 to -0,004467	0,0002
GRN vs. SNr	0,005488	-0,002438 to 0,01341	0,3898
GRN vs. APN	0,003477	-0,004449 to 0,01140	0,8806
GRN vs. SCm	0,003015	-0,004911 to 0,01094	0,9430
VM vs. SNr	0,01788	0,009955 to 0,02581	<0,0001
VM vs. APN	0,01587	0,007944 to 0,02380	<0,0001
VM vs. SCm	0,01541	0,007482 to 0,02333	<0,0001
SNr vs. APN	-0,002011	-0,009937 to 0,005915	0,9954
SNr vs. SCm	-0,002473	-0,01040 to 0,005453	0,9823
APN vs. SCm	-0,0004614	-0,008387 to 0,007465	>0,9999

Supplementary Table 3. P values global pathology on contralateral hemisphere

Comparison	Mean Diff.	95% CI of diff.	Adjusted P value
CEA vs. BST	-0,07093	-0,09630 to -0,04555	<0,0001
CEA vs. SNc	-0,06371	-0,08908 to -0,03833	<0,0001
CEA vs. LC/PB	0,03946	0,01409 to 0,06484	0,0002
CEA vs. GRN	0,05239	0,02702 to 0,07777	<0,0001
CEA vs. VM	0,02025	-0,005125 to 0,04562	0,2156
CEA vs. SNr	0,03960	0,01423 to 0,06497	0,0002
CEA vs. APN	0,03738	0,01201 to 0,06275	0,0006
CEA vs. SCm	0,04788	0,02251 to 0,07326	<0,0001
BST vs. SNc	0,007221	-0,01815 to 0,03259	0,9901
BST vs. LC/PB	0,1104	0,08501 to 0,1358	<0,0001
BST vs. GRN	0,1233	0,09794 to 0,1487	<0,0001
BST vs. VM	0,09118	0,06580 to 0,1165	<0,0001
BST vs. SNr	0,1105	0,08515 to 0,1359	<0,0001
BST vs. APN	0,1083	0,08293 to 0,1337	<0,0001
BST vs. SCm	0,1188	0,09344 to 0,1442	<0,0001
SNc vs. LC/PB	0,1032	0,07779 to 0,1285	<0,0001
SNc vs. GRN	0,1161	0,09072 to 0,1415	<0,0001
SNc vs. VM	0,08395	0,05858 to 0,1093	<0,0001
SNc vs. SNr	0,1033	0,07793 to 0,1287	<0,0001
SNc vs. APN	0,1011	0,07571 to 0,1265	<0,0001
SNc vs. SCm	0,1116	0,08621 to 0,1370	<0,0001
LC/PB vs. GRN	0,01293	-0,01244 to 0,03830	0,7666
LC/PB vs. VM	-0,01921	-0,04459 to 0,006161	0,2755
LC/PB vs. SNr	0,0001388	-0,02523 to 0,02551	>0,9999
LC/PB vs. APN	-0,002082	-0,02746 to 0,02329	>0,9999
LC/PB vs. SCm	0,008421	-0,01695 to 0,03379	0,9741
GRN vs. VM	-0,03214	-0,05752 to -0,006769	0,0045
GRN vs. SNr	-0,01279	-0,03817 to 0,01258	0,7767
GRN vs. APN	-0,01501	-0,04039 to 0,01036	0,5994
GRN vs. SCm	-0,004510	-0,02988 to 0,02086	0,9996
VM vs. SNr	0,01935	-0,006023 to 0,04472	0,2669
VM vs. APN	0,01713	-0,008244 to 0,04250	0,4238
VM vs. SCm	0,02763	0,002260 to 0,05301	0,0236
SNr vs. APN	-0,002221	-0,02759 to 0,02315	>0,9999
SNr vs. SCm	0,008282	-0,01709 to 0,03366	0,9766
APN vs. SCm	0,01050	-0,01487 to 0,03588	0,9107

Supplementary Table 4. P values global pathology on ipsilateral hemisphere
